# Recombinant Bivalent Fusion Protein rVE Induces CD4+ and CD8+ T-Cell Mediated Memory Immune Response for Protection Against *Yersinia enterocolitica* Infection

**DOI:** 10.3389/fmicb.2015.01407

**Published:** 2015-12-16

**Authors:** Amit K. Singh, Joseph J. Kingston, Shishir K. Gupta, Harsh V. Batra

**Affiliations:** ^1^Department of Microbiology, Defence Food Research Laboratory, Defence Research and Development OrganisationMysore, India; ^2^Department of Bioinformatics, Biocenter, University of WürzburgWürzburg, Germany

**Keywords:** recombinant protein rVE, CD4+ T cells, CD8+ T cells, cytokine profiling, memory immune responses, *Yersinia enterocolitica*

## Abstract

Studies investigating the correlates of immune protection against *Yersinia* infection have established that both humoral and cell mediated immune responses are required for the comprehensive protection. In our previous study, we established that the bivalent fusion protein (rVE) comprising immunologically active regions of *Y. pestis* LcrV (100–270 aa) and YopE (50–213 aa) proteins conferred complete passive and active protection against lethal *Y. enterocolitica* 8081 challenge. In the present study, cohort of BALB/c mice immunized with rVE or its component proteins rV, rE were assessed for cell mediated immune responses and memory immune protection against *Y. enterocolitica* 8081. rVE immunization resulted in extensive proliferation of both CD4 and CD8 T cell subsets; significantly high antibody titer with balanced IgG1: IgG2a/IgG2b isotypes (1:1 ratio) and up-regulation of both Th1 (TNF-α, IFN-γ, IL-2, and IL-12) and Th2 (IL-4) cytokines. On the other hand, rV immunization resulted in Th2 biased IgG response (11:1 ratio) and proliferation of CD4+ T-cell; rE group of mice exhibited considerably lower serum antibody titer with predominant Th1 response (1:3 ratio) and CD8+ T-cell proliferation. Comprehensive protection with superior survival (100%) was observed among rVE immunized mice when compared to the significantly lower survival rates among rE (37.5%) and rV (25%) groups when IP challenged with *Y. enterocolitica* 8081 after 120 days of immunization. Findings in this and our earlier studies define the bivalent fusion protein rVE as a potent candidate vaccine molecule with the capability to concurrently stimulate humoral and cell mediated immune responses and a proof of concept for developing efficient subunit vaccines against Gram negative facultative intracellular bacterial pathogens.

## Introduction

The human pathogenic *Yersinia* species, *Y. enterocolitica* and *Y. pseudotuberculosis* are enteric pathogens, whereas *Y. pestis* is the causative agent of acute zoonotic disease plague (Brubaker, 1991; Bottone, 1997; Perry and Fetherston, 1997). Empirical studies aimed to develop vaccine molecules against *Yersinia* infection have established that concerted humoral and cell mediated immunity coupled with long lived memory response is required for the comprehensive protection (Parent et al., 2005, 2006; Philipovskiy and Smiley, 2007). The specific antibodies elicited by humoral immunity neutralizes extracellular bacteria or their virulence factors whereas, clearance of intracellular bacterial pathogens largely depends upon T-cell response through cytokine production. T-cell-dependent cellular immunity comprises another means by which vaccines can prime long-lived memory protection characterized with heightened and faster development of effector cells and specific antibodies upon subsequent encounter with the same pathogen/antigen.

Live attenuated plague vaccine strains elicit protective immunity against the disease in humans and derivatives of Girard and Robic’s EV76 attenuated strain, had been licensed for human use in Soviet Union and China (Feodorova and Motin, 2012; Feodorova et al., 2014). However, fatalities reported in small animals including non-human primates (Meyer, 1970; Meyer et al., 1974; Russell et al., 1995), reactogenicity manifested in human vaccines and constraints in (post exposure) prophylactic co-administration of antibiotics among risk population prelude their wide spread application (Meyer et al., 1974; Welkos et al., 2002). In addition, the emerging antibiotic resistant *Y. pestis* strains raise recurring zoonosis concern in modern world endemic regions and their possible employment as a potential bio-warfare agent (Galimand et al., 1997; White et al., 2002; Williamson and Oyston, 2012; Lister et al., 2012). These factors highlight the need to develop protective subunit vaccines against *Y. pestis* infection.

In order to develop safe and effective candidate subunit vaccines, tremendous efforts have been made by various research groups but confined mostly to *Y. pestis* LcrV, and F1 (Overheim et al., 2005; Chichester et al., 2009; Quenee et al., 2011) proteins that largely rely upon CD4 Th-2 antibodies for protection in different animal models (Parent et al., 2005; Williamson et al., 2005). On the other hand, YopE a T3SS protein conserved among pathogenic *Yersinia* provided CD8 Th1 cells mediated protection in C57BL6 mice (Lin et al., 2011; Zhang et al., 2012). Considering the necessity of the plague vaccines to generate both humoral and cellular immunity for comprehensive protection (Philipovskiy and Smiley, 2007), we designed a recombinant bivalent fusion protein rVE encompassing immunologically active regions of *Y. pestis* LcrV and YopE proteins. Immunization with rVE protein developed robust humoral immune response in mice and provided comprehensive protection while its component proteins could not provide complete protection when administered individually (Singh et al., 2014). This made us speculate that the comprehensive protection exhibited by the bivalent fusion molecule could be due to the cumulative effect of both humoral and cell mediated immune responses. The present investigation was therefore taken up to estimate cell mediated and memory immune responses elicited by rVE and its component proteins. Vaccination with purified bivalent protein rVE developed CD4+ and CD8+ T cells mediated cellular immune protection dominated with proinflammatory cytokines. The cellular immune responses were capable of maintaining immune protection for more than four months from the day of final immunization.

## Materials and Methods

### *In Silico* Structure Prediction of Bivalent Fusion Protein rVE

*In silico* structure prediction of truncated recombinant proteins rV and rE derived from LcrV (100–270 aa) and YopE (50–213 aa.), respectively, of *Y. pestis* and their fusion construct (rVE) was performed by I-TASSER (Roy et al., 2010) implemented composite modeling approach which includes the identification of suitable templates, reassembly of fragment structure, building of atomic models and selection of the best model (Zhang, 2009). The quality of the predicted protein structures by I-TASSER was estimated by confidence score (Zhang, 2008) that is based on the significance of the threading template alignments and the convergence parameters of the I-TASSER’s structure assembly refinement simulations. Furthermore, to correct the stereochemistry and to eliminate bad contacts between protein atoms and structural water molecules, the models were solvated and subjected to constraint energy minimization with a harmonic constraint of 100 kJ/mol/Å^2^ using the steepest descent and conjugate gradient technique by using the GROMOS96 43B1 force field (Scott et al., 1999) parameters set implemented in SwissPdb Viewer (Kaplan and Littlejohn, 2001). The quality assessment of the refined protein models were performed by Psi/Phi inspection of the Ramachandran plot obtained from PROCHECK analysis (Laskowski et al., 1993).

### Bacterial Strain and Infection

The *Y. enterocolitica* 8081 (O:8 serotype) used in this study was kindly provided by Dr. J. S. Virdi (University of Delhi, South Campus). For protection studies, the overnight grown culture of *Y. enterocolitica* 8081 was freshly inoculated in tryptone soya broth (TSB) broth supplemented with 20 mM MgCl_2_ and kept in shaker incubator at 28°C until OD reached 0.5–0.8 at 600 nm. Two hours before infection, the grown culture was shifted to 37°C for induction of Type III Secretion System (T3SS). Culture was washed and resuspended in phosphate buffer saline (PBS) containing 10% iron dextran (Robins-Browne and Prpic, 1985).

For preparing whole cell lysates, bacterial cells were disrupted by sonication using 5–6 pulses for 30 s at 100 Watts (Hielscher ultrasonic processor, Germany), on ice. Soluble whole cell protein was collected by centrifugation at 12000 rpm at 4°C for 10 min followed by passage through 0.2 μm syringe filter and stored at –80°C until further application (Hoy et al., 2012).

### Ethics Statement

All animal management and research procedures were conducted under animal use protocols approved by the Institutional Animal Ethical Committee, Defence Food Research Laboratory (DFRL/28/IAEC/CPCSEA), completely accredited by Committee for the Purpose of Control and Supervision of Experiments on Animals (CPCSEA), India. During the course of experiment, mice were maintained in a pathogen free facility and fed with sterile food and water *ad libitum*. Mice vaccination or challenge were performed under anesthesia induced by intra peritoneal (IP) injection with 200 μl of 0.5% xylazine and 0.2% ketamine mixture (200:1) and all possible efforts were imposed to minimize sufferings.

### Mice and Immunization

Female BALB/c mice of 6–8 weeks age were procured from Central Animal Facility, Defence Food Research Laboratory (DFRL), Mysore. All animals were maintained in pathogen free condition in accordance with the Institutional Animal Ethical Committee (DFRL, Mysore) norms. Groups of eight mice were IM (intra-muscular) immunized into the hind legs with 50 μg individual truncated recombinant proteins (rV and rE) and their fusion protein (rVE) mixed with aluminum hydrogel (1:1 ratio) at an interval of 14 days (0, 14, 28, and 42 day) (Overheim et al., 2005). Sera were collected bi-weekly from retro-orbital sinuses by competently trained technical staff in a hygienic condition. Mice were anesthetized through the IP route with 200 μl of 0.5% xylazine and 0.2% ketamine mixture (200:1) and individual blood collections were managed upto maximum 10% of the total blood volume from each mice (Uppada et al., 2011).

### Antibody Titer and Isotypes

After 42 days of scheduled immunization, the specific antibody titers were determined by indirect enzyme-linked immunosorbent assay (ELISA) as described previously by Singh et al. (2014). In brief, serially diluted test sera were added in triplicate on the 96 well microtiter plates coated with recombinant proteins (1 μg/well). Bound antibodies were detected by reacting with peroxidase conjugated goat anti-mouse polyvalent antibodies (Sigma, India). The specific antibody titer in test sera was estimated by maximum serum dilution with OD (450 nm) at least twofold greater than sham immunized mice (Uppada et al., 2011; Singh et al., 2014).

Antigen specific immunoglobulin subclasses IgM, IgG1, IgG2a, IgG2b, and IgG3 were assessed by using mouse isotyping kit (ISO-2KT, Sigma India) by following manufacturer’s protocol for indirect ELISA. Briefly, sera obtained after 42nd day immunization were serially diluted in carbonate-bicarbonate buffer (pH 9.2 ± 0.2) and added into antigen coated (1 μg/well) microtiter plates. After 1 h of incubation, diluted sera was replaced with goat anti-mouse isotype specific monoclonal antibodies (1:1000 diluted in PBS) followed by 1 h incubation with polyvalent rabbit anti-goat HRP conjugate. Antibody isotype titers were estimated similar to serum antibody titer.

### Lymphocyte Proliferation Assay

Spleen was collected aseptically from euthanized, ex-sanguinated experimental mice and homogenized in Dulbecco’s Modified Eagle’s Medium (DMEM) to recover the splenocytes. The splenocytes were pelleted by centrifugation at 800 × *g* for 5 min at 37°C and the erythrocytes were lysed by incubation with RBC lysis buffer pH 7.2 ± 0.2 (155 mM ammonium chloride, 10 mM potassium bicarbonate and 127 μM EDTA) at 37°C for 3–5 min followed by two washes in sterile PBS. The splenocytes in DMEM media (1 × 10^6^ cells/ml) was distributed in 96 well culture plates and incubated at 37°C with 5% CO_2_. Gradient concentration of rV, rE, and rVE recombinant proteins (5, 10, 15, 20, 25, and 30 μg/ml) were added in lymphocyte suspension and incubated at 37°C (5% CO_2_) for 72 h (Leary et al., 1995; Sun et al., 2014). Concanavalin-A (Con A) at 20 μg/ml concentration was used as a positive control. Proliferation of lymphocytes were assessed by incubating with 50 μg of MTT [3-(4,5-dimethythiazol-2-yl)-2,5-diphenyl tetrazolium bromide] reagent in dark for 2 h at 37°C. Reaction was developed with 200 μl of dimethyl sulfoxide (DMSO) and observed at 570 nm in plate reader (Infinite M200 pro; Tecan, Grodig, Austria). All the supplements and media were procured from Sigma, India.

### Cytokine Estimation

The pro-inflammatory cytokines (IL-2, IL-12, IFN-γ, TNF-α) and anti-inflammatory cytokines (IL-4, IL-10) elicited in immunized (rV, rE, and rVE) and sham immunized groups of mice were examined using sandwich ELISA based cytokine estimation kit (Mabtech, Nacka, Sweden) by following manufacturer’s instructions. In brief, 96 well microtiter plates coated with capture monoclonal antibodies were incubated with culture supernatants from splenocytes exposed with recombinant proteins (30 μg/ml) or cell lysate of *Y. enterocolitica* 8081 10^6^ cells, followed by biotin-labeled detection antibodies for 2 h at 37°C. Each well was washed 5–6 times with PBST (PBS with 0.05% Tween 20) and incubated with Streptavidin- HRP conjugate. Reaction was developed with 50 μl substrate *O*-phenylenediaminedihydrochloride (OPD) in the presence of 0.4% H_2_O_2_ and absorbance was measured in ELISA plate reader (Infinite M200 pro; Tecan, Grodig, Austria) at 450 nm. Cytokine standards were included in triplicates in each assay, and were used for the calculation of cytokines level in test samples.

### Flow Cytometric Analysis of CD4+ and CD8+ T-Cell Immune Response

Anti-coagulated blood (EDTA 2 mM) obtained from immunized and sham immunized mice groups 24 h post challenge were used for CD4+ and CD8+ T-cell estimation. The blood samples were pooled and erythrocytes lysed with 1:10 diluted lysis solution (FACS lysing solution, BD) for 30 min in dark at 37°C. After 30 min incubation, cells were harvested, washed twice with Dulbecco’s PBS and 10^6^ cells were stained with rat-anti-mouse FITC-labeled anti-CD3, APC-labeled anti-CD4, and PE-labeled anti-CD8 monoclonal antibodies (BD Biosciences). A minimum of 20,000 events were counted for each analysis using BD Accuri^TM^ C6 Flow cytometer (Becton-Dickinson) and results were analyzed using Kaluza software version 3.1v (Beckman Coulter, USA). The percent activated CD3+ T cells were electronic gated among total lymphocytes obtained and CD4+ and CD8+ of T cells population in the gated CD3+ T cells was determined (Williamson et al., 2005).

### Animal Challenge

*Y. enterocolitica* 8081 was grown in TSB broth with 20 mM MgCl_2_, washed and diluted in sterile PBS supplemented with 10% iron dextran solution and IP challenged (10^8^ CFU/mice) (Robins-Browne and Prpic, 1985). Mortality and morbidity were recorded for 24 days post challenge. On 2, 7, 14, and 21 days of challenge, one mouse from each group was injected with 200 μl of 0.5% xylazine and 0.2% ketamine mixture (200:1) and humanely killed to censor the health status and Colony Forming Unit (CFU) count in spleen and liver (Overheim et al., 2005). The survival percentage in each group of challenged mice was calculated by using Kaplan Meier method.

### Statistical Analysis

All the experiments were repeated thrice with similar conditions. Results were presented as the means value ± standard deviation (SD). Survival curves were constructed by Kaplan–Meier method using GraphPad Prism (v5.02). Statistical difference between various treatment groups were analyzed by uni-variate/One way ANOVA using Dunnett’s and Tukey’s *post hoc* test.

## Results

### Computational Modeling of Protein Structure

**FIGURE 1:**
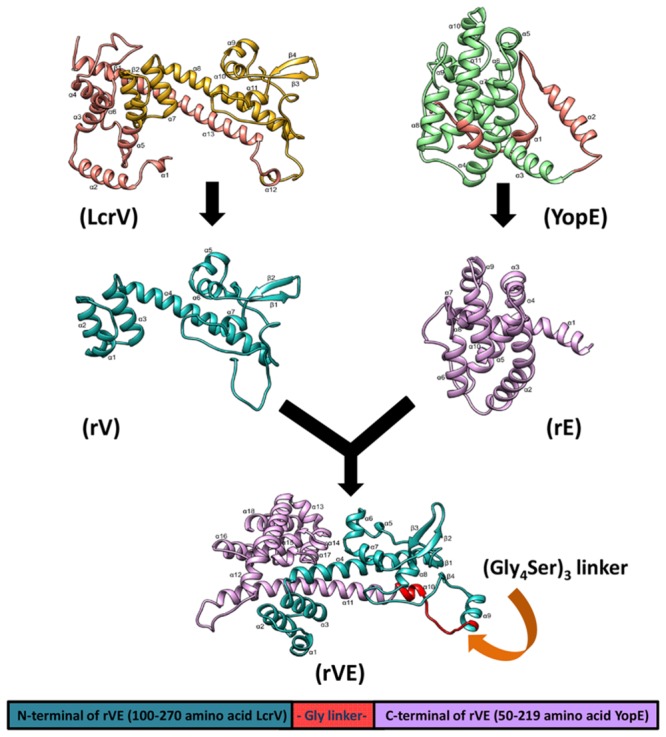
**The three dimensional predicted structure of rVE fusion protein as ribbon model, was determined by I-TASSER composite modeling approach.** The N-terminal domain (rV) and C- terminal domain (rE) are colored in green and violet, respectively, with omitted regions represented as brown color in both native structures (LcrV and YopE). The recombinant rV and rE components of rVE bivalent fusion protein attached by a flexible glycine linker presented in red color.

To assess whether the antigenic targets presented by the fusion protein resemble the native component proteins we predicted their 3D structures. The TM-score and C-score calculated for the predicted models revealed correct modeling of recombinant proteins rV, rE and rVE (Supplementary Tables S1 and S2). The stereo chemical quality assessment of the refined protein models were performed by inspection of the Psi/Phi Ramachandran plot obtained from PROCHECK. In all the models, >90% residues were present in core regions (Supplementary Table S3). This indicated that the backbone dihedral angles, Psi and Phi in the models were reasonably accurate. The 3D structure of modeled proteins is depicted in **Figure 1** showed that the recombinant protein structures were very similar and compared with native proteins.

### Immunization with rVE Elicited Both Th1 and Th2 IgG Isotypes

**FIGURE 2:**
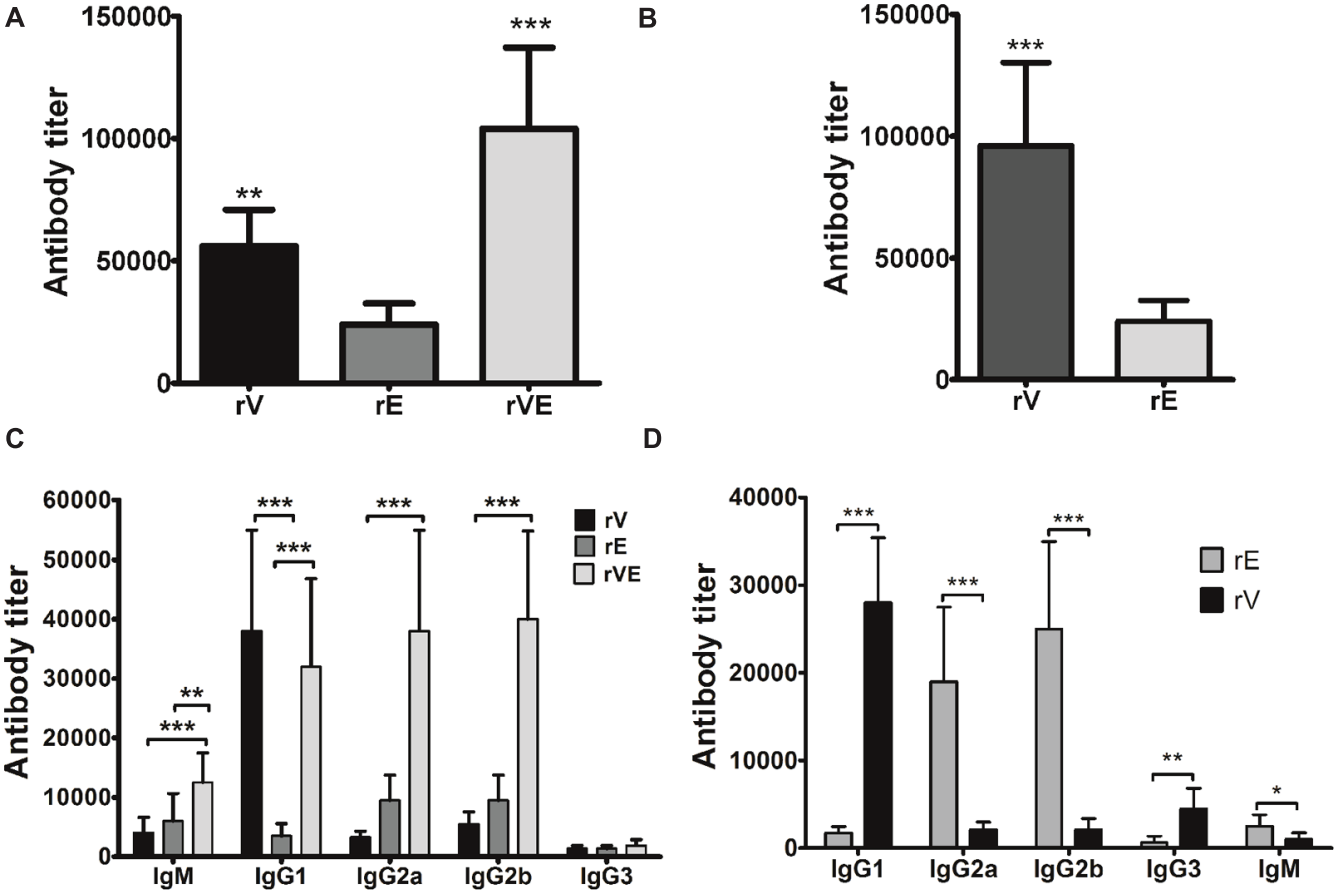
**Specific antibody response and antibody isotype profiling estimated by end point titers of murine polysera raised in immunized mice. (A)** Total serum anti-rV, anti-rE and anti-rVE antibody titers assessed after 42nd day of immunization. **(B)** The proportion of rV and rE specific serum antibody titers in rVE immunized mice after final immunization at 42nd day. **(C)** Specific antibody subclasses in sera of BALB/c mice immunized with recombinant protein rV, rE, and rVE at 43rd day. **(D)** rV and rE specific antibody subclasses in rVE immunized mice. Results are expressed as antibody titer, the maximum dilution of serum having OD twofolds higher than sham mice polysera. The data are representing as mean value ± SD (*n* = 8). ^∗^*P* < 0.05; ^∗∗^*P* < 0.01; ^∗∗∗^*P* < 0.001.

After 42 days of scheduled immunization, the group of mice immunized with rV and rVE exhibited significantly higher antibody titer (5.6 × 10^4^ and 1.04 × 10^5^, respectively) than rE (2.4 × 10^4^) (**Figure 2A**). Immunization with rVE developed fourfold higher serum antibody titer against rV component than that of rE (**Figure 2B**). The IgG antibody subclasses and IgM level specific to respective recombinant proteins were analyzed from sera samples obtained after final immunization (43rd day). Mice vaccinated with rV and rE elicited prominent IgG1 (3.8 × 10^4^) and IgG2a, IgG2b (9.5 × 10^3^ each), respectively, whereas, rVE elicited enhanced response of both IgG1 (3.2 × 10^4^) and IgG2a, IgG2b (3.8 × 10^4^, 4.0 × 10^4^) antibodies (**Figure 2C**). The antigenic competitive assay performed by using rVE, rV and rE proteins against anti-rVE polysera revealed the development of IgG1 and IgG2a/IgG2b antibody titer against rV and rE components, respectively, in bivalent fusion protein (rVE) immunized mice (**Figure 2D**). The IgM and IgG3 isotype titers were insignificant in any of the sera (**Figure 2C**).

### *In Vitro* Proliferation of Lymphocyte

**FIGURE 3:**
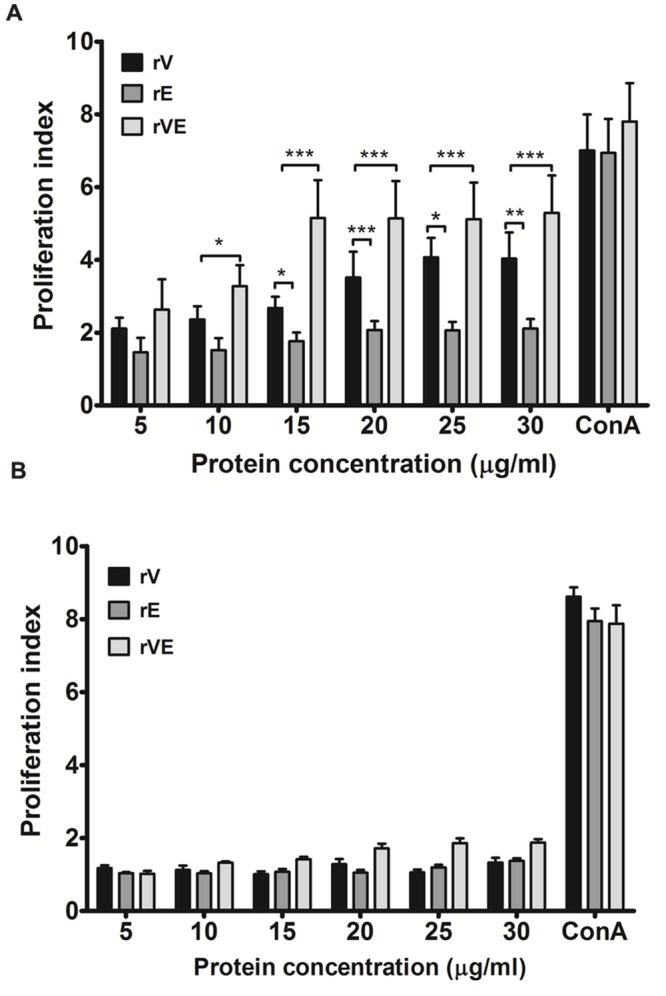
**Proliferation assay performed with lymphocytes isolated from the spleen of immunized and sham immunized groups of mice at 43rd day. (A)** Lymphocytes isolated from the rV, rE, and rVE immunized mice were cultured in 96-well flat bottom plates with a gradient concentration of purified recombinant proteins. After 72 h of incubation at 37°C (5% CO_2_), proliferative responses were determined by MTT assay. **(B)** Proliferation assay performed with lymphocytes obtained from sham immunized mice spleen and were stimulated with gradient concentration of purified recombinant proteins (rV, rE, and rVE). The T-cell inducer, ConA at 20 μg/ml was considered as positive control. Extracted lymphocytes with culture medium alone were used as a negative control. Data represented as PI mean values ± SD. ^∗^*P* < 0.05; ^∗∗^, *P* < 0.01; ^∗∗∗^, *P* < 0.001; compared to control group (sham immunized mice).

Splenocytes obtained from immunized (rV, rE, and rVE) and sham immunized group of mice were examined for their ability to proliferate in presence of gradient concentration of recombinant proteins with ConA (20 μg/ml) as positive control. A significantly higher (*P* < 0.001) proliferation was observed in rVE primed splenocytes at 15 μg/ml concentration of rVE protein [Proliferation index (PI) value 5.7], with no significant increase in PI value with further protein concentration. The rV and rE induced splenocytes were observed with lower PI values of 4.071 and 2.062, respectively, without further increase in PI values after 25 μg/ml (**Figure 3A**). The sham immunized mice splenocytes showed no significant change in PI value when induced with any of the proteins (**Figure 3B**).

### Induction of Th1 and Th2 Cytokines and Inhibition of LcrV Mediated Immunomodulatory Response by rVE Immunization

**FIGURE 4:**
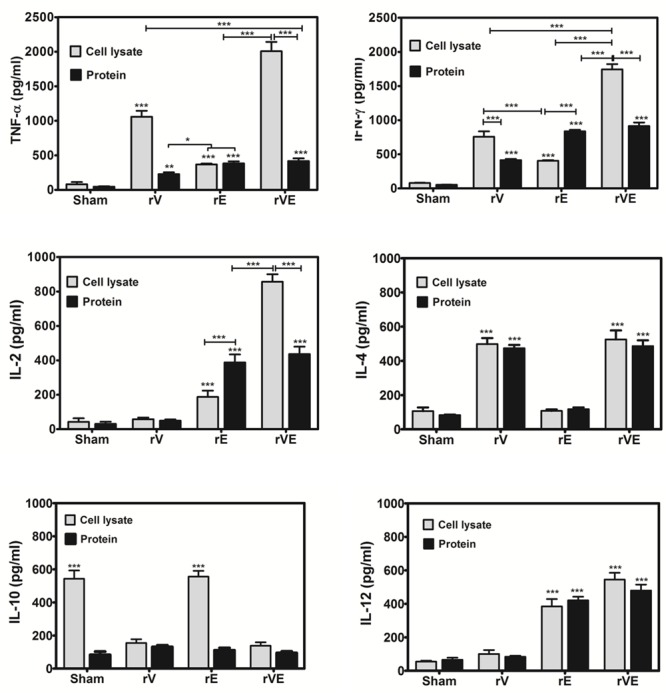
**Cytokine profile in mice immunized with recombinant proteins rV, rE, and rVE.** Splenocytes isolated from immunized BALB/c mice at 43rd day after initial immunization were stimulated *in vitro* with 30 μg/ml of respective recombinant purified proteins or whole cell lysate of 10^6^ *Y. enterocolitica*. The sham immunized mice splenocytes stimulated with purified proteins (rV, rE, and rVE) or whole cell lysate were considered as negative controls. Levels of pro-inflammatory (IFN-γ, TNF-α, IL-2, IL-12) and anti-inflammatory (IL-4, IL-10) cytokines in the culture supernatants produced from splenocytes after 72 h of incubation were measured by Mabtech ELISA kit. Data represent the mean ± SD of the results determined. ^∗^*P* < 0.05; ^∗∗^*P* < 0.01; ^∗∗∗^*P* < 0.001.

Splenocytes from mice immunized with rV, rE, and rVE were exposed to the respective proteins and production of pro-inflammatory (TNF-α, IFN-γ, IL-2, IL-12) or anti-inflammatory cytokines (IL-4, IL-10) were estimated from culture supernatant of primed splenocytes (**Figure 4**). Significantly higher levels of pro-inflammatory (TNF-α, IFN-γ, IL-2, and IL-12) and anti-inflammatory (IL-4) cytokines were observed in rE and rV primed splenocytes, respectively. Splenocytes from mice primed with rVE exhibited significantly (*P* < 0.001) higher levels of both pro and anti-inflammatory cytokines while IL-10 was not observed in any of the groups. In another set of experiment the cytokines levels were measured from antigen primed splenocytes exposed to *Y. enterocolitica* whole cell lysate. IL-10 was observed in control and rE primed splenocytes and not in other mice groups. The expression of pro-inflammatory cytokines (IL-2 and IFN-γ) in rE group which was on par with rVE when stimulated with their respective recombinant proteins. However, when stimulated with whole cell lysate, the expression of pro-inflammatory cytokines in rE group were significantly (*P* < 0.001) lower than similar cytokines elicited by rVE. Substantially higher levels of the pro-inflammatory cytokines TNF-α and IFN-γ were observed in rV and rVE groups, *in vitro* stimulated with *Y. enterocolitica* cell lysate. The IL-4 and IL-12 levels did not vary significantly when stimulated either with recombinant proteins or whole cell lysate (**Figure 4**).

### Immunization with rVE Elicits CD4+ and CD8+ T-Cells Mediated Protective Memory Responses

**FIGURE 5:**
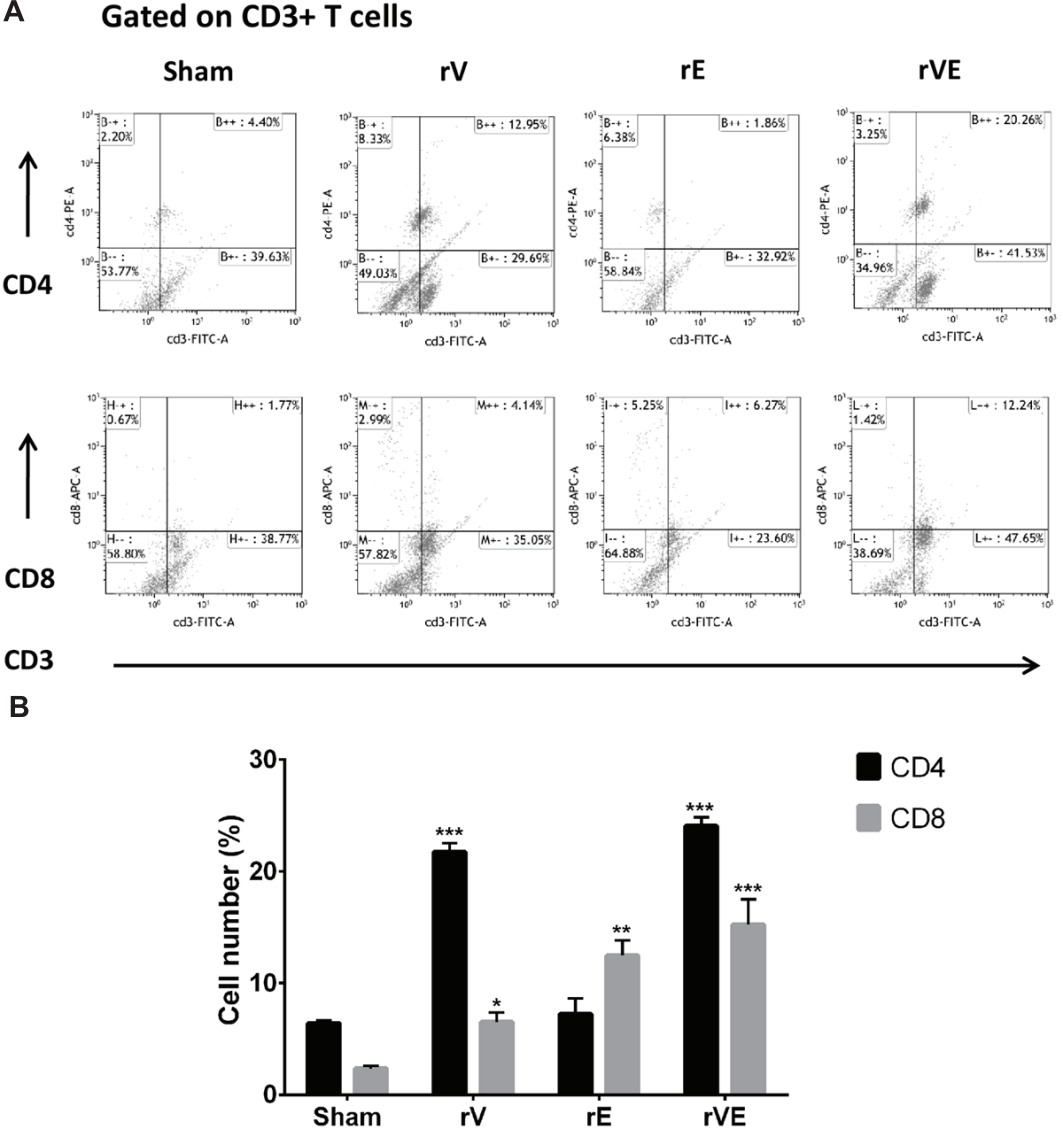
**The CD4 and CD8 T-cells were estimated by flow cytometric analysis using fluorescent tagged anti-CD3, anti-CD4, and anti-CD8 monoclonal antibodies.** PBMCs isolated from three representative rV, rE, and rVE immunized mice after 24 h of IP challenge with *Y. enterocolitica* 8081 were used in the assay. **(A)** Representative graphs showing percentage of CD4 and CD8 T cells within gated CD3 T cell population. The percentage of gated T cells in each quadrant is shown. **(B)** The percentage CD4 and CD8 T cell population in immunized and sham immunized mice. ^∗^*P* < 0.05; ^∗∗^*P* < 0.01; ^∗∗∗^*P* < 0.001 compared to sham immunized mice CD4 and CD8 T cell population.

**FIGURE 6:**
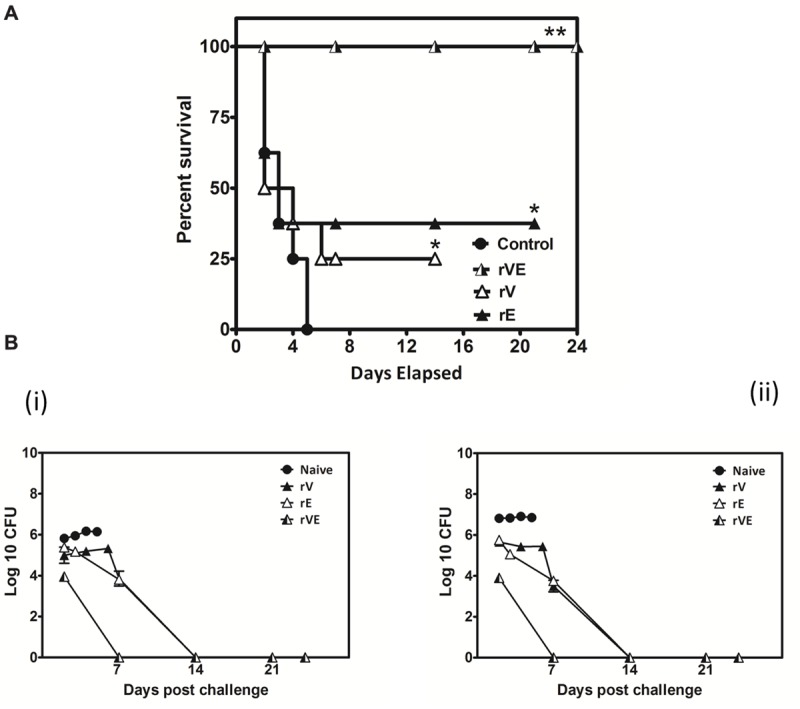
**Actively vaccinated mice and sham vaccinated mice were challenged with *Y. enterocolitica* 8081 to assess the memory immune protection. (A)** Group of BALB/c mice scheduled immunized for 42nd day with 50 μg of recombinant proteins (rV, rE, and rVE) and left uninfected for 120 days. After incubation of 120 days mice were IP injected with 10^8^ CFU of *Y. enterocolitica* and survival was monitored for 24 days. Sham immunized mice were considered as control. Data represented as the percentage survival calculated by Kaplan–Meier method using Graphpad Prism5. **(B)** Bacterial burden in immunized and sham immunized mice challenged with *Y. enterocolitica* 8081. The analysis of total Colony Forming Units (CFU) in liver (i), spleen (ii), were performed at different time points (day 2, 7, 14, and 24) post challenge. Data pooled from 2 to 4 biological replicates in two statistical repeats were analyzed by Univariate method applying Dunnett’s and Tukey’stest. ^∗^*P* < 0.05; ^∗∗^*P* < 0.01; ^∗∗∗^*P* < 0.001.

The blood samples collected from immunized and sham immunized groups of mice were analyzed for the generation of CD4+ and CD8+ T-cells. The flow cytometric (FC) analysis showed predominant generation of CD4+ and CD8+ T-cells in mice immunized with rV and rE proteins, respectively, whereas co-expression of both the T-cell subtypes CD4+ and CD8+ T-cells was observed in rVE immunized mice (**Figure 5**). The sham immunized group of mice showed no significant proliferation of CD4+ or CD8+ T-cells (**Figure 5**). The rVE immunized mice were completely protected with 100% survival when challenged with 10^8^ CFU of *Y. enterocolitica* 121st day post incubation (**Figure 6A**). About 10^4^ CFU were observed in both liver and spleen on 2nd day after challenge and the bacterial burden was cleared by 7th day (**Figure 6Bi,ii**). Significant bacterial colonization was observed in liver and spleen of rV and rE groups of mice which resulted in 6/8 and 5/8 mice succumbing to infection within 6 and 3 days with 25 and 37.5% survival, respectively (**Figure 6A,B**). The control mice group was observed with higher bacterial colonization in liver and spleen (10^6^ and 10^5^, respectively) at 2nd day of infection followed with gradual increase in CFU count till 5th day. The excessive bacterial burden led to tissue necrosis with hepatomegaly and splenomegaly conditions (Data not shown) and all mice succumbed to infection within 5 days.

## Discussion

The human pathogenic *Yersinia* species (*Y. pestis, Y. pseudotuberculosis*, and *Y. enterocolitica*) are facultative intracellular pathogen (Brubaker, 1991; Bottone, 1997; Perry and Fetherston, 1997). At early stages of infection they survive and multiply within the host epithelial and macrophage cells however, predominantly stay extracellular replicating within the necrotic foci (Janssen and Surgalla, 1969; Cowan et al., 2000; Pujol and Bliska, 2003, 2005; Lukaszewski et al., 2005). Vaccine study against *Y. pestis* have extensively focused upon the protective antibodies elicited by subunit vaccine molecules especially, *Y. pestis* capsular F1 protein and T3SS component LcrV (Parent et al., 2005; Philipovskiy and Smiley, 2007; Smiley, 2008). The convalescent sera against F1-V used in serotherapy was protective in mice against lethal *Y. pestis* pulmonary infection (Hill et al., 2006; Fellows et al., 2010) however, failed to completely protect the cohort of F1-V immunized non-human primates against aerosol preparation of *Y. pestis*, despite high antibody titer (Bashaw et al., 2007). The antibodies provide protection against pulmonary *Y. pestis* infection only if there is a significant generation of TNF-α and IFN-γ (Parent et al., 2005, 2006; Lin et al., 2011) thus proving that both antibodies and cytokines play role in protection via distinguishable, independent pathways (Kummer et al., 2008). Previously, we had generated a fusion protein rVE encompassing immunodominant regions of *Y. pestis* LcrV (rV) and YopE (rE) separated by a glycine linker; it was highly immunogenic, generated high titer protective antibodies and provided 100% passive and active protection against lethal challenge of *Y. enterocolitica* 8081. The present work was undertaken to assess the prevalence of CD4 and CD8 T cell epitopes, in the rV and rE components derived from *Y. pestis* LcrV and YopE proteins, respectively, and whether these two main T lymphocyte subsets could be triggered when rVE fusion protein is used for immunization. Further the ability of the fusion protein to balance Th1 and Th2 polarization was examined by studying the IgG isotype and cytokine patterns and these immunological correlates were compared with the long term protection achieved in mice groups against *Y. enterocolitica* 8081 challenge.

The *in silico* structure prediction of individual recombinant proteins (rV and rE) and their fusion construct rVE by I-TESSER server implemented composite modeling revealed that combining rV and rE protein together through a glycine linker (Gly_4_Ser)_3_ achieved characteristic 3D structure, comparative to the native full length LcrV and YopE proteins. Mice immunized with rV exhibited significantly higher Th2 biased IgG response featuring 11:1 ratio of IgG1:IgG2a/IgG2b while, rE group of mice were observed with considerably lower serum antibody titer with predominant Th1 response (IgG1:IgG2a/IgG2b 1:3 ratio), consistent with previously reported findings (Chichester et al., 2009; Lin et al., 2011). On the other hand rVE immunized group elicited higher antibody titer fractionated with IgG1:IgG2a/2b subclasses in 1:1 ratio signifying balanced activation of both Th2 and Th1 immune responses. Fusion protein thus negated the inadequate Th2 and Th1 responses associated with YopE and LcrV, respectively.

Splenocytes from mice primed with the recombinant proteins exhibited significantly higher proliferation index at low protein concentration indicating the immunogenic nature of all recombinant proteins. Flow cytometric analysis of T-cell population revealed higher proliferation of CD4 and CD8 subsets among mice groups immunized with truncated rV and rE proteins, respectively, and was strikingly similar to that reported for the entire individual proteins earlier (Parent et al., 2005; Lin et al., 2011; Zhang et al., 2012). Extensive proliferation of both the T cell subsets were observed in mice immunized with the fusion protein rVE which provided functional proof that component epitopes were maintained intact in the 3D structure of the fusion protein rVE during protein refolding. Recent studies to determine the role of T cell subsets in providing protection against *Y. pestis* infection have proved that both CD4 and CD8 cells are required for comprehensive protection whereas, either of them could not provide complete protection individually (Parent et al., 2005; Philipovskiy and Smiley, 2007). While the CD8 T cells provide the effector arm to the cell-mediated immune response, CD4 T cells influence their optimal maturation and differentiation from naïve CD8 T cells into cytotoxic effector and memory cells (Swain et al., 2006). The ability of bivalent protein to prime both CD4 and CD8 T cells qualifies it as an effective subunit vaccine candidate.

The rV (21 kDa) protein devoid of the 31–57 and 270–300 aa regions of native LcrV (37 kDa) that are otherwise responsible for TLR2 mediated IL-10 induction, resulted in low level of IL-10 expression in primed splenocytes induced with the recombinant protein (rV). On the other hand, higher levels of IL-10 were seen in rE and sham immunized mice when primed splenocytes were inoculated with whole cell lysate of *Y. enterocolitica* while it remained depressed in rV and rVE groups. This up regulation of IL-10 could be possibly due to the immunomodulatory role played by native LcrV from whole cell lysate which in turn down regulated the expression of pro-inflammatory cytokine IL-2 and IFN-γ in rE groups. Whereas, no significant down regulatory effects were observed for the expression of TNF-α and IL-12. We thus speculate that the down regulation of IL-10 seen in rV and rVE immunized mice splenocytes inoculated with whole cell lysate could be due to the rV component’s ability to counter the native LcrV mediated immunomodulatory function (Nedialkov et al., 1997; Overheim et al., 2005; Sing et al., 2005). Higher levels of IL-2 and IL-12 cytokines help proliferation of Type I helper cells (Th1) and the associated effector cytokines TNF-α and IFN-γ, that in turn assists to eliminate intracellular infection are considered indispensable for protection against *Y. pestis* infection (Parent et al., 2005, 2006). Both IL-2 and IL-12 were conspicuous in rVE immunized mice. IL-2 facilitates longer survival of antigen stimulated CD4+ T cells while IL-12 is required for the acquisition of cytolytic property by antigen stimulated CD8 T cells and their optimal IL-2 dependent proliferation and clonal expansion (Valenzuela et al., 2002; Schmidt et al., 2008). IL-4 that helps differentiation of Th2 cells was also up-regulated in the rVE group without any reciprocal inhibition by IFN-γ usually reported otherwise (Yao et al., 2005). The concerted induction of both CD4+ and CD8 + T cell types and balanced up-regulation of both Th1 (TNF-α, IFN-γ, IL-2, and IL-12) and Th2 (IL-4) cytokines in rVE group of mice correlated with their superior survival rates (100%) than rE (37.5%) and rV (25%) groups of mice upon challenge after 120 days of immunization. The bacterial colonization in liver and spleen of rVE immunized mice was significantly lesser than other groups of mice and the bacterial burden was cleared in 7 days. The correlates of immune response associated with protection against *Y. pestis* infection could be observed in our study and this correlated with protection against *Y. enteroclolitica* infection. The core pathogenicity of yersiniae largely rely upon the 70 kb plasmid encoded Yop virulon to subvert host immune responses (Brubaker, 1991; Cornelis, 2002). Despite difference in the routes of entry, and disease manifestation, the pathogenic yersiniae retain almost equal affinity for the lymphoid tissues and subvert the function of host macrophages and neutrophils (Brubaker, 1991; Pujol and Bliska, 2005). This commonality in Yop virulon which includes LcrV and YopE molecules among pathogenic yersiniae could be the reason for the cross protection observed in our study.

Findings in this and our earlier studies define the bivalent fusion protein rVE as a potent candidate vaccine molecule with the capability to concurrently stimulate humoral and cell mediated immune responses; CD4, CD8 T cell subsets and provide 100% protection. The rV protein derived from LcrV was devoid of the immunomodulatory property otherwise associated with the entire protein but possessed CD4+ and Th2 response receptors. Similarly the rE region chosen possessed CD8+ and Th1 response receptors and possibly helped improve the memory response along with the rV component. We could not explain the reason for the increased expression of pro-inflammatory cytokines IL-2, TNF-α, and IFN-γ in rVE splenocytes stimulated with *Y. enterocolitica* whole cell lysate than that of recombinant protein, which needs to be elucidated subsequently.

## Author Contributions

Experiments carried out in the study were performed by AS. Whereas, *in silico* studies and 3D predication of recombinant and native proteins structures was done by SG. AS, JK, and HB were involved in designing of experiments, data interpretation, and manuscript preparation.

## Conflict of Interest Statement

The authors declare that the research was conducted in the absence of any commercial or financial relationships that could be construed as a potential conflict of interest.
