# Supplementary material for: Recombinant Bivalent Fusion Protein rVE Induces CD4+ and CD8+ T-Cell Mediated Memory Immune Response for Protection Against Yersinia enterocolitica Infection
Source: Front Microbiol. 2015 Dec 16;6:1407. doi: 10.3389/fmicb.2015.01407 (PMC4679870; doi:10.3389/fmicb.2015.01407)
Supplement: Supplementary file 1 [file Data_Sheet_1.DOCX]

**Supplementary Table 1:** C-alpha root mean square deviation (RMSD) between the protein pairs

.

| **Protein pairs** | **C-alpha root mean square deviation (RMSD)** |
| --- | --- |
| LcrV and recombinant LcrV (LcrV rV) | 1.0 Å |
| YopE and recombinant YopE (YopE rE) | 1.6 Å |
| LcrV and Fusion protein (LcrV_YopE_rVE) | 2.0 Å |
| YopE and Fusion protein (LcrV_YopE_rVE) | 1.2 Å |

**Supplementary Table 2:** Estimated Template modeling score (TM-score) and confidence score (C-score) for the predicted protein structures

| **Protein models** | **TM-score** | **C-score** |
| --- | --- | --- |
| Native LcrV | 0.68±0.12 | -0.26 |
| rV | 0.88±0.07 | 1.19 |
| Native YopE | 0.38±0.13 | -2.95 |
| rE | 0.44±0.14 | -2.38 |
| rVE | 0.56±0.15 | -1.28 |

**Supplementary Table 3:** Ramachandran plot statistics of modeled proteins obtained from PROCHECK analysis.

| **Protein** | **Residues in most favoured region [a,b,l]** | **Residues in additional allowed Region [a,b,l,p]** | **Residues in generously allowed region [~a,~b,~l,~p]** | **Residues in disallowed region** |
| --- | --- | --- | --- | --- |
| LcrV | 89.8% | 7.5% | 1.6% | 1.0% |
| rV | 90.1% | 6.8% | 1.0% | 2.1% |
| YopE | 86.5% | 11.4% | 2.2% | 0.0% |
| rE | 83.1% | 9.6% | 3.0% | 4.2% |
| rVE | 92.4% | 5.1% | 0.9% | 1.5% |
